# X-ray characterization of Ge dots epitaxially grown on nanostructured Si islands on silicon-on-insulator substrates

**DOI:** 10.1107/S0021889813003518

**Published:** 2013-06-07

**Authors:** Peter Zaumseil, Grzegorz Kozlowski, Yuji Yamamoto, Markus Andreas Schubert, Thomas Schroeder

**Affiliations:** a IHP, Im Technologiepark 25, Frankfurt (Oder), 15236, Germany; b Brandenburgische Technische Universität Cottbus, Konrad-Zuse-Strasse 1, Cottbus, 03046, Germany

**Keywords:** nanostructured Si, Ge heteroepitaxy, silicon-on-insulator (SOI) substrates, grazing-incidence X-ray diffraction, transmission electron microscopy (TEM)

## Abstract

On the way to integrate lattice mismatched semiconductors on Si(001), the Ge/Si heterosystem was used as a case study for the concept of compliant substrate effects that offer the vision to be able to integrate defect-free alternative semiconductor structures on Si. Ge nanoclusters were selectively grown by chemical vapour deposition on Si nano-islands on silicon-on-insulator (SOI) substrates. The strain states of Ge clusters and Si islands were measured by grazing-incidence diffraction using a laboratory-based X-ray diffraction technique. A tensile strain of up to 0.5% was detected in the Si islands after direct Ge deposition. Using a thin (∼10 nm) SiGe buffer layer between Si and Ge the tensile strain increases to 1.8%. Transmission electron microscopy studies confirm the absence of a regular grid of misfit dislocations in such structures. This clear experimental evidence for the compliance of Si nano-islands on SOI substrates opens a new integration concept that is not only limited to Ge but also extendable to semiconductors like III–V and II–VI materials.

## Abstract 2

Selective epitaxial growth of Ge on nanostructured Si islands on silicon-on-insulator substrates is investigated by X-ray diffraction and transmission electron microscopy to prove the compliance effect between the materials and the structural perfection, especially under the use of a thin SiGe buffer layer.

## 1. Introduction

The integration of alternative semiconductor materials on silicon is an important materials science approach to increase performance and/or functionality of Si microelectronics. InGaAs heterostructures have been investigated as high-mobility n-channel materials for scaled complementary metal oxide semiconductor (CMOS) transistors (Sun *et al.*, 2007 ▶), and Ge photonic modules (*e.g.* ultra-fast photodetectors) have been integrated in Si CMOSs to merge electronics and photonics on a single Si chip (DeRose *et al.*, 2011 ▶).

Advanced selective heteroepitaxy techniques to enable lattice mismatched semiconductor integration on Si(001) are thus of fundamental importance. The Ge/Si system is probably the most extensively investigated material combination. The integration of Ge may be done either globally over the whole Si wafer to set up so-called virtual Ge substrates on Si or locally by selective growth techniques to limit Ge thin-film deposition to the area of the future device.

Owing to the large lattice mismatch between Ge and Si of about 4.2%, a global deposition of Ge on Si will be accompanied unavoidably by the formation of misfit dislocations at the interface. Therefore, much effort has been made to reduce at least the number of threading dislocations that are responsible for the degradation of electrical properties of Ge. Thick graded SiGe buffer layers were introduced (Currie *et al.*, 1998 ▶), the direct deposition of Ge on Si was optimized by annealing steps during/after the Ge layer growth (Hartmann *et al.*, 2005 ▶; Choi *et al.*, 2008 ▶; Yamamoto *et al.*, 2011 ▶), heteroepitaxially grown rare earth oxides like Pr_2_O_3_ were used as a buffer layer on blanket Si wafers (Giussani *et al.*, 2009 ▶), or patterned Si(001) substrates with growth windows in SiO_2_ masks were used (Fitzgerald, 1989 ▶; Park *et al.*, 2007 ▶).

An alternative approach to the classical thin-film deposition is selective Ge growth on nanostructured Si substrates. The basics of this so-called nano-heteroepitaxy (NHE) were developed by Zubia & Hersee (1999 ▶) and Zubia *et al.* (2000 ▶). The nanostructured substrate acts as a compliant material that accommodates part of the misfit strain of the epilayer by an elastic response on the growing film. Under ideal conditions, this may result in a film growth completely free of misfit dislocations.

In our previous studies we tried to apply the NHE approach to the Ge/Si system. A chemical-vapour-deposition-based process was used to grow Ge selectively on Si nano-pillars of different dimensions (Zaumseil *et al.*, 2011 ▶; Kozlowski *et al.*, 2011 ▶, 2012 ▶). Clear compliance behaviour of the Si substrate was not achieved in these experiments, mainly owing to the SiO_2_ growth mask, which is necessary to protect the side walls of the Si nanostructures and interspaces from unwanted Ge deposition. A weak improvement was obtained by the use of thin SiGe buffer layers (Zaumseil, Kozlowski, Schubert *et al.*, 2012 ▶). In a second approach we investigated nanostructured Si islands on silicon-on-insulator (SOI) substrates by advanced third-generation synchrotron radiation techniques and found clear experimental evidence for the compliance of Si nano-islands (Zaumseil, Kozlowski, Yamamoto *et al.*, 2012 ▶).

In this paper we demonstrate that the strain characterization of nanostructured Si islands on SOI substrates with two-dimensional periodic arrangement is also possible by the use of a laboratory-based X-ray diffraction technique only. We show in a ‘proof of principle’ study that by the use of ∼10 nm-thick Si_(1−*x*)_Ge_*x*_ buffer layers the compliance behaviour of the Si islands can be significantly improved up to about 1.8% tensile strain, which is nearly half the misfit value between Ge and Si.

## 2. Experimental details

SOI wafers of (001) orientation and 200 mm diameter with 54 nm box Si and 145 nm SiO_2_ were used for the preparation of Si nano-islands with Ge dots on top. The thickness of the box Si of some wafers was reduced to 27 nm by wet oxidation and HF etching. Standard procedures of lithographic structuring followed by dry etching were used to generate a two-dimensional pattern of Si islands of different diameter with 360 nm periodicity. The free-standing SiO_2_ thickness is reduced in this process by ∼37 nm. The side walls of the Si islands were cleaned by growth of 10 nm SiO_2_, which was removed afterwards by etching.

**Figure 1:**
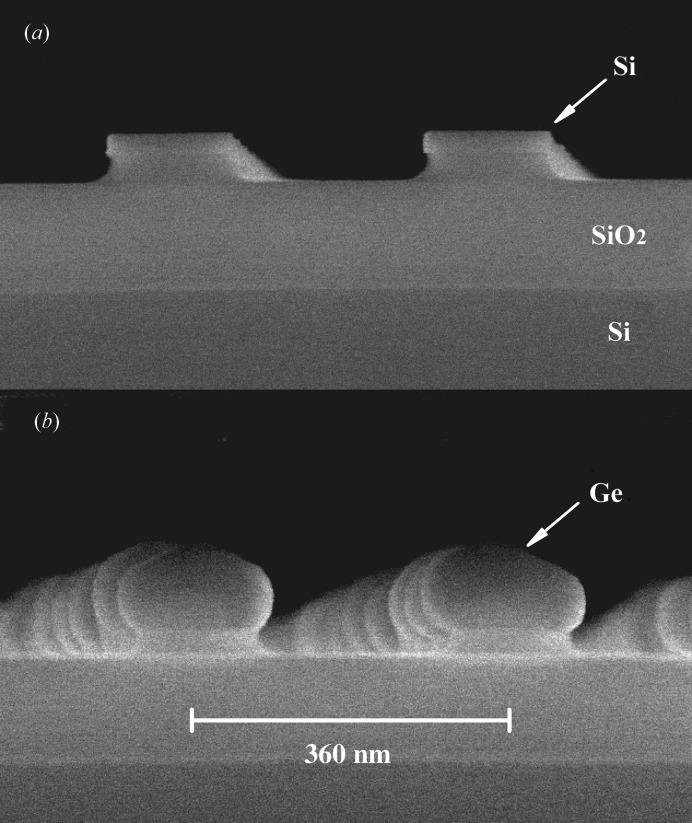
SEM micrographs of two samples with 27 nm-thick Si islands of 125 nm diameter on 145 nm SiO_2_ in the pre-deposited state (*a*) and after Ge deposition and annealing at 1023 K (*b*).

Fig. 1 ▶(*a*) shows a typical scanning electron microscopy (SEM) image of ∼125 nm-diameter Si islands sitting on 40 nm-high SiO_2_ mesa structures after nitride removal and final cleaning. After a short prebake at 1123 K in a reduced-pressure chemical vapour deposition reactor, Ge was deposited either directly on the Si islands by a two-step process, with a Ge seed layer grown at 573 K followed by the Ge growth process at 823 K, or on 10 nm-thick Si_(1−*x*)_Ge_*x*_ buffer layers deposited at 873 K with nominal Ge content *x* = 0.6 and 0.3. The calibration of the Ge deposition was done on blanket bulk Si wafers so that the real values for structured SOI wafers might be different. Post-deposition annealing was performed at 973, 1023 or 1073 K for 1 min in an H_2_ atmosphere. Fig. 1 ▶(*b*) shows a SEM micrograph after direct Ge deposition and annealing at 1023 K.

All of the grazing-incidence X-ray diffraction (GID) measurements were performed with a Rigaku SmartLab diffractometer equipped with a 9 kW rotating anode using Cu *K*α radiation. This diffractometer allows a sample to be kept in a horizontal position during the measurements. An automatic alignment procedure is used to adjust the sample surface normal parallel to the ϕ rotation axis of the diffractometer with an accuracy of about 0.002°. This guarantees a well defined and constant angle of incidence independent of the sample in-plane orientation. A Ge(400) two-crystal collimator uses the Cu *K*α_1_ wavelength only and reduces the beam divergence in the direction of specular diffraction to about 0.003°. The divergence of the incident beam in the direction of the GID diffraction is limited by a 0.5° Soller slit, while a 0.114° Soller slit system in front of the detector determines mainly the angular resolution of the total arrangement. The incoming beam has a horizontal width of 5 mm and a height of 0.1 mm. The diffracted beam is measured with 5 mm horizontal width and 3 mm open vertical slits (integral scheme in exit angle α_f_). The obtained resolution is 0.08° FWHM for a ϕ rotation and 0.15° for a 2θ/ϕ rotation for the 400 reflection of a perfect Si sample. For absolute measurements of in-plane lattice parameters, the angular scale of the diffractometer was always checked (calibrated) with the signal of the Si substrate.

SEM images were obtained with an S4500-II from Hitachi, and transmission electron microscopy (TEM) studies were carried out using an FEI Tecnai Osiris instrument.

## 3. Results and discussion

**Figure 2:**
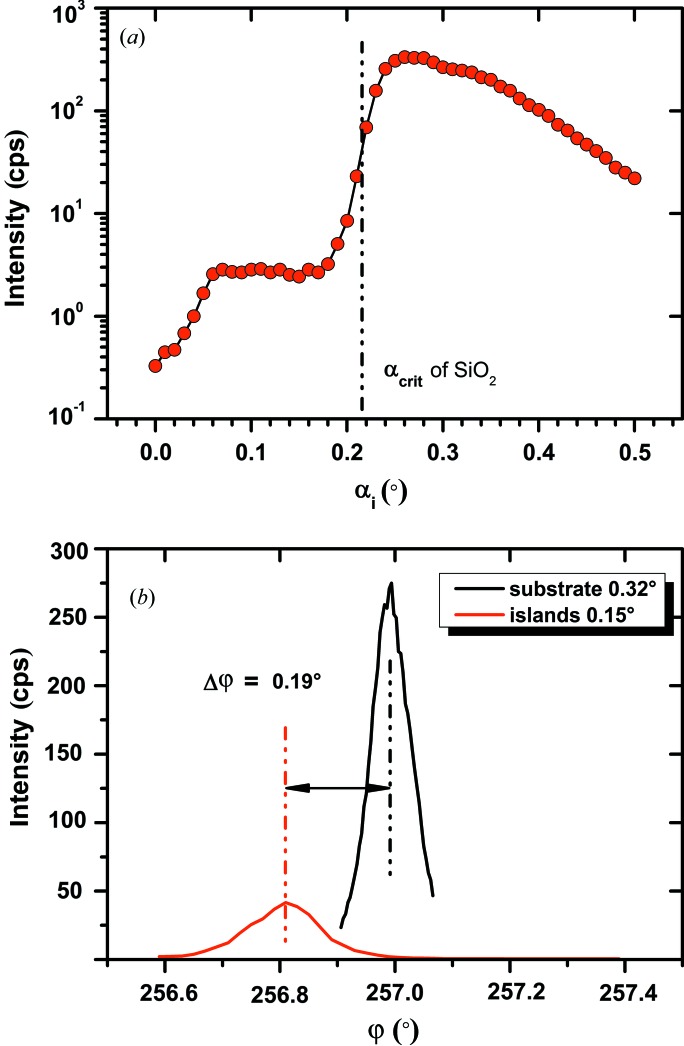
(*a*) Intensity of in-plane Si 400 diffraction *versus* angle of incidence α_i_ for a sample with Si islands only and (*b*) its dependence on in-plane orientation ϕ for Si substrate and islands.

The strain state characterization of the Si nano-islands, which is one of the main goals of this study, requires a clear separation between the signals from Si islands and Si substrate. Two features are of advantage in this respect, as demonstrated in Fig. 2 ▶. Taking the diffracted intensity of the in-plane 400 reflection as a function of the angle of incidence α_i_, the Si islands on top of the used material stack contribute from low angles on, while the Si substrate gives a clear signal above the critical angle of the SiO_2_ layer of about 0.22° only. Furthermore, the crystal lattices of the Si substrate and the Si box layer do not have exactly the same in-plane orientation. Scans in ϕ (ϕ axis is parallel to the [001] substrate lattice normal) at the same Bragg angle but α_i_ values suited for Si islands and substrate, respectively, confirm a misorientation of some tenth of a degree, with slight differences for individual wafers. Thus it is possible to be especially sensitive to the Si islands by selection of proper ϕ and α_i_ values. On the other hand, the Si substrate signal can be used to calibrate the diffractometer.

The in-plane lattice parameter *a*_0_ determined from the peak position of the 400 Si island reflection for structures without Ge deposition is, within the error limits, very close to the bulk Si value for all investigated samples. Calculated strain values ∊_0_ = (*a*_0_ − *a*_Si_)/*a*_Si_ vary between −0.02 and +0.11%.

**Figure 3:**
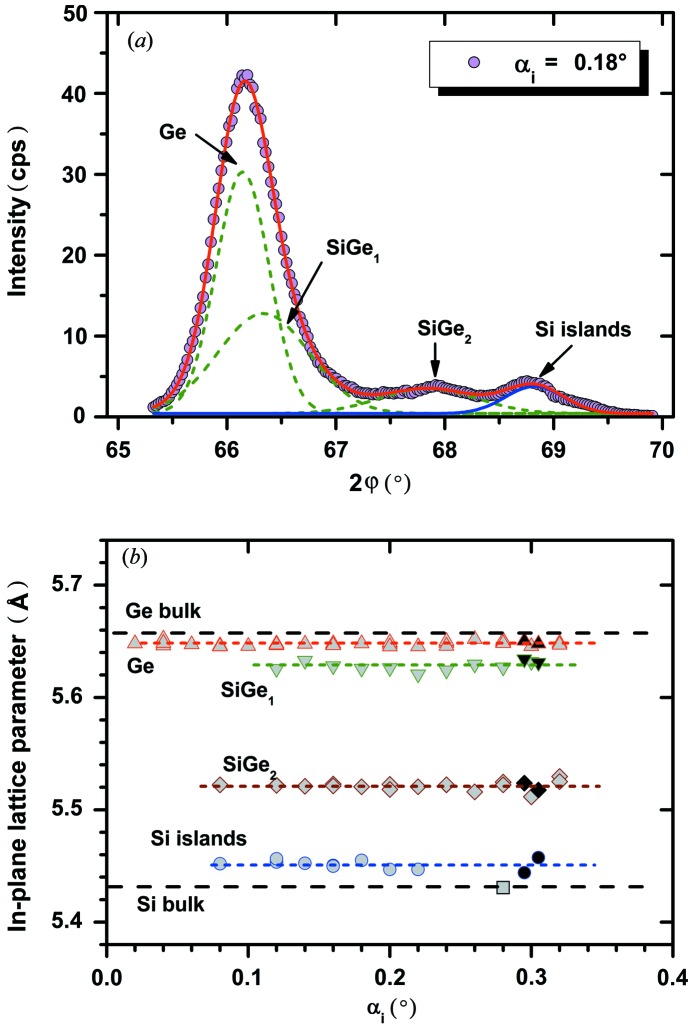
(*a*) In-plane 400 diffraction curve at α_i_ = 0.18° and its fitting by four Gaussian profiles and (*b*) the estimated in-plane lattice parameters for similar fittings at different α_i_ values. The data shown with black filled symbols were obtained by synchrotron measurements of 400 and 620 diffraction for comparison.

Fig. 3 ▶(*a*) shows a typical 2θ/ϕ scan of the 400 reflection for a sample with Si islands of about 125 nm diameter and 54 nm thickness with Ge directly deposited on it in the as-deposited state. Similar curves were measured for incident angles between 0.02 and 0.32°. In this case, the curve can be fitted by four Gaussian profiles that are marked as Ge, SiGe_1_, SiGe_2_ and Si islands.

The in-plane lattice parameters calculated from the corresponding peak position are plotted as a function of α_i_ in Fig. 3 ▶(*b*). The results of different measurements are in very good agreement, and the averaged values of such a set of data increase the accuracy and reliability significantly compared with a single measurement. The highest signal is related to the Ge dot. Supposing that the diffracting material is pure Ge, which was confirmed by specular 004 measurements (not shown), the slightly smaller in-plane lattice parameter compared with the Ge bulk value indicates a compressive strain of about −0.17%. The next peak (SiGe_1_) is obviously the result of fitting the slightly asymmetric Ge peak within the four-curve model and can be explained by two mechanisms: (i) the in-diffusion of Si that forms an SiGe alloy with high Ge content and/or (ii) the existence of stronger compressively strained parts of the Ge dots.

The pronounced SiGe_2_ peak indicates the existence of a significant volume fraction in the grown Ge that has formed an SiGe alloy with a relative constant Ge content of about 42%. Owing to possible additional strain components, this value is only a rough estimate. The fact that this peak is well separated from the Ge/SiGe_1_ peak on one side and the Si islands peak on the other side shows that there does not exist a smooth transition of Si content from the Si islands to the pure Ge as would be expected for a thermal diffusion process. This fragment of SiGe volume might be the result of a strain-driven interdiffusion process in the early stages of Ge growth.

**Figure 4:**
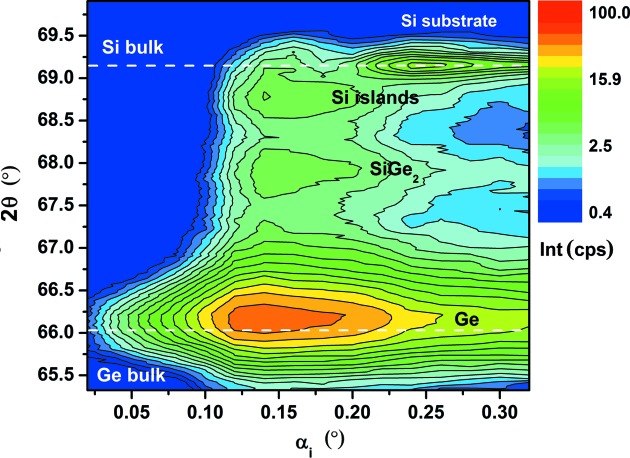
Intensity of in-plane 400 diffraction *versus* 2θ and α_i_ of the same sample as in Fig. 3 ▶.

**Figure 5:**
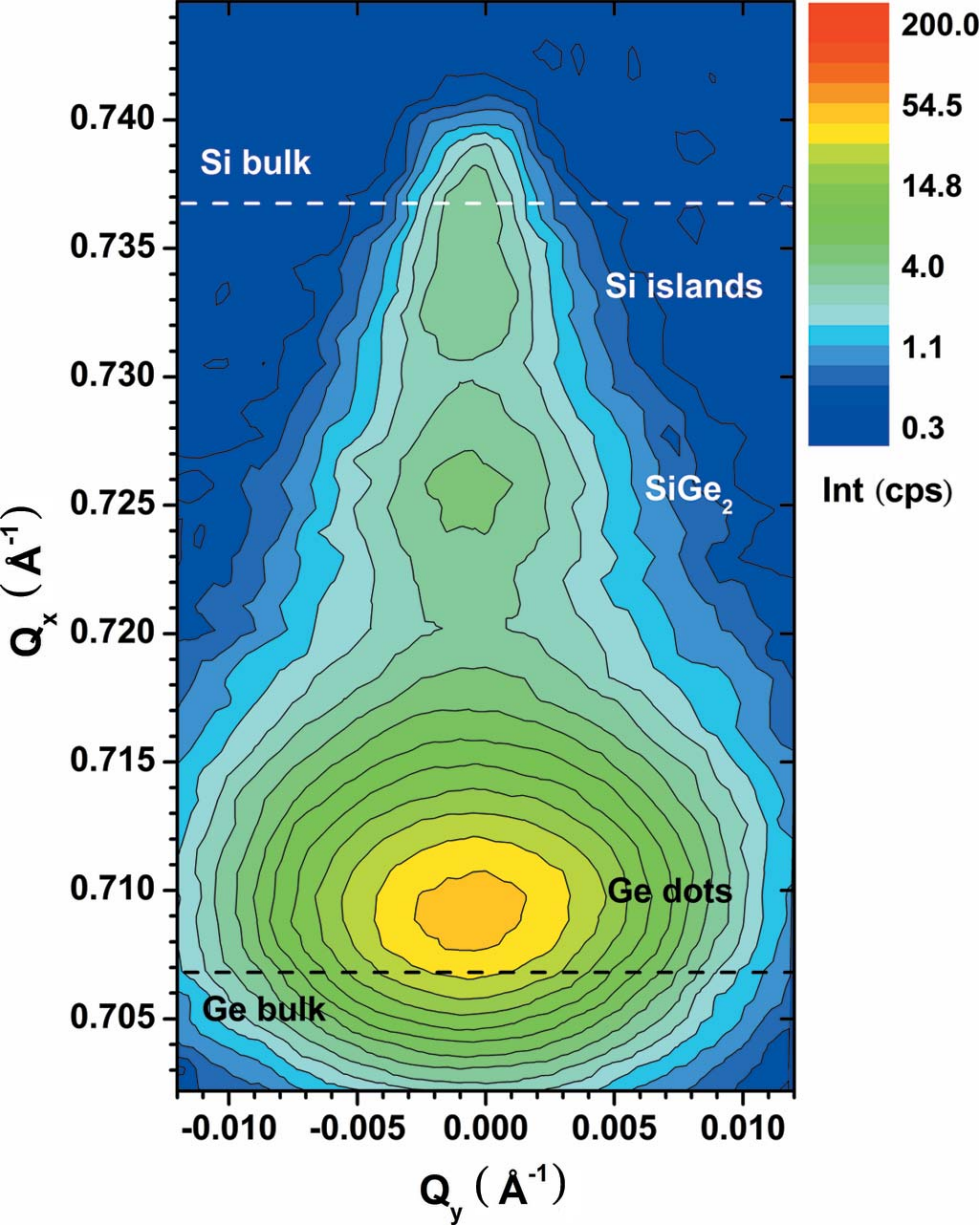
Reciprocal space map of 400 diffraction of the same sample as in Figs. 3 ▶ and 4 ▶ measured at α_i_ = 0.18°.

The in-plane lattice constant calculated from the Si islands peaks is 5.452 Å, which correlates to a tensile strain of the Si islands of +0.39%. It was demonstrated by synchrotron energy dispersive X-ray diffraction studies (Zaumseil, Kozlowski, Yamamoto *et al.*, 2012 ▶) that the Si islands of such structures remain pure Si and no detectable Ge in-diffusion takes place. Thus, this is a clear proof that nanostructured Si islands on SOI substrates show a compliance effect after direct deposition of Ge. Fig. 4 ▶ demonstrates in a two-dimensional plot of 400 diffraction intensity *versus* 2θ and α_i_ that the signals of Si islands and Si substrate can be well separated for this sample. This kind of plot allows an optimal selection of α_i_ values for the measurement of a reciprocal space map (RSM) as shown in Fig. 5 ▶.

In addition to the laboratory-based measurements, the results of 400 and 620 measurements performed at beamline ID1 at ESRF (Zaumseil, Kozlowski, Yamamoto *et al.*, 2012 ▶) are shown in Fig. 3 ▶(*b*) for comparison, which demonstrates the very good agreement between both measuring techniques. A similar good agreement is shown in a comparison of the RSM obtained with a laboratory-based technique (Fig. 5 ▶) with an RSM measured at ID1 [see Fig. 2 ▶ of Zaumseil, Kozlowski, Yamamoto *et al.* (2012 ▶)].

Comparable investigations of samples with different Si island geometries show that the tensile strain can be varied to some extent. A sample with similar Ge deposition conditions and island diameter to those of the sample used for Fig. 3 ▶, but with 27 nm island thickness only, shows a tensile strain of +0.52%. Additional annealing leads to the tendency of decreasing strain.

The tensile strain observed for samples with Ge directly deposited on Si nano-islands is relatively small compared with the 4.2% misfit between Ge and Si and suggests that there are still misfit dislocations generated at the Ge/Si interface to realize the nearly full relaxation of the Ge lattice. This can be understood by the growth dynamics of this system, where a non-compliant phase must always be passed through before the compliance effect works. For Ge growth on Si the critical thickness to form misfit dislocations is only a few nanometres, and plastic relaxation occurs before sufficient strain energy is built up to strain the Si island.

The balance between the critical thickness to form misfit dislocations and the strain partitioning in nanostructures can be tailored for the Ge/Si system by the use of Si_(1−*x*)_Ge_*x*_ with suitable Ge content *x* (Zubia *et al.*, 2000 ▶). The positive effect of SiGe buffer layers concerning a compliance effect on nanostructured bulk Si substrates has already been described for Si nano-pillars (Zaumseil, Kozlowski, Schubert *et al.*, 2012 ▶).

**Figure 6:**
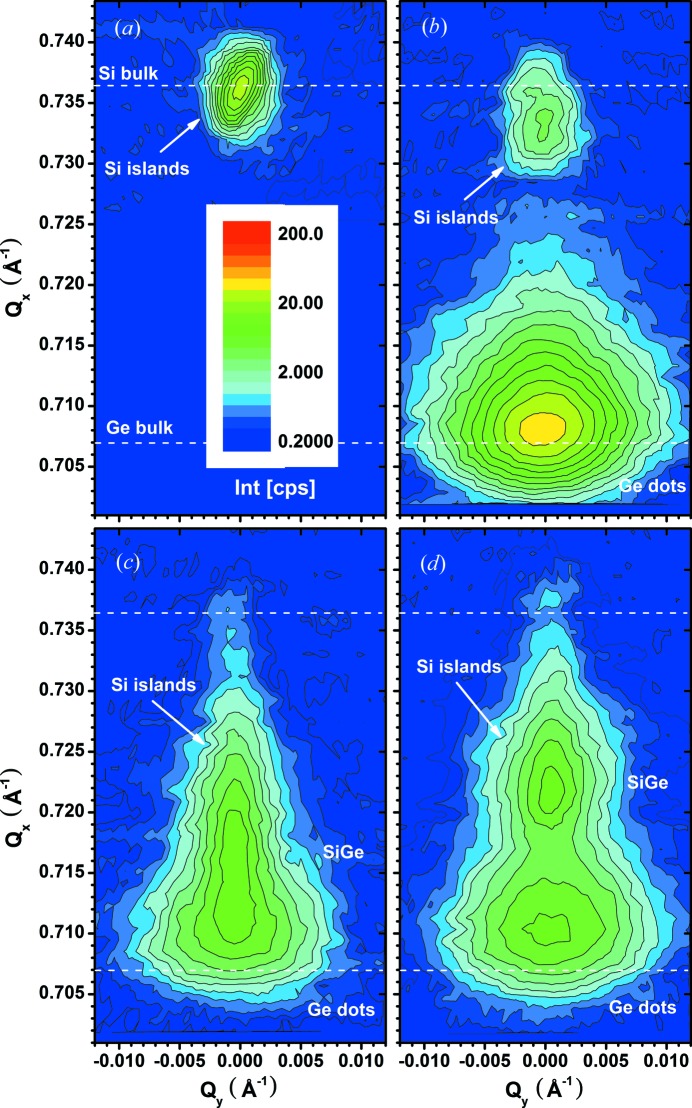
Comparison of reciprocal space maps of samples: (*a*) Si islands only, (*b*) after direct Ge deposition, (*c*) after Ge deposition on an Si_0.4_Ge_0.6_ buffer layer and (*d*) after Ge deposition on an Si_0.7_Ge_0.3_ buffer layer in the as-deposited state measured at α_i_ = 0.12°.

Fig. 6 ▶ shows a set of RSMs with 27 nm-thick Si islands (*a*), after direct Ge deposition (*b*), and after Ge deposition on Si_0.4_Ge_0.6_ (*c*) and Si_0.7_Ge_0.3_ (*d*) buffer layers in the as-deposited state. The position of the Si islands in the uncovered state agrees well with the expected position for bulk Si. After direct Ge deposition it shifts slightly towards the Ge spot to smaller *Q*_*x*_ values, indicating a tensile in-plane strain. The RSM of each sample with an SiGe buffer is more complex. A separated Si island signal is no longer visible. Most probably it has shifted further to smaller *Q*_*x*_ values and been superimposed by the SiGe buffer signal.

**Figure 7:**
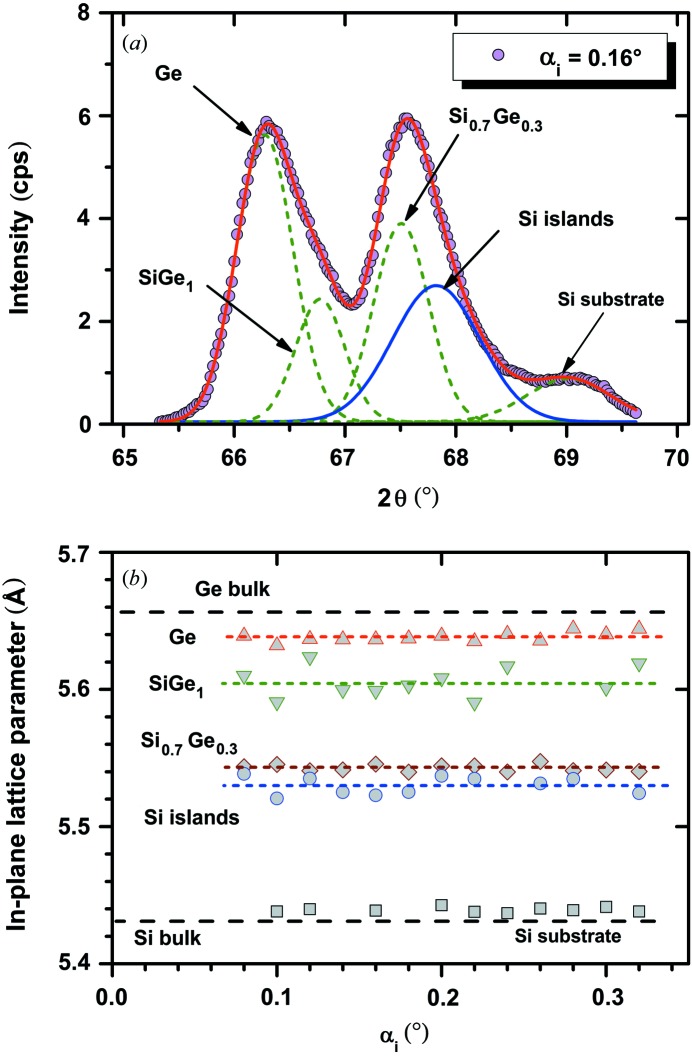
In-plane 400 diffraction curve at α_i_ = 0.16° of a sample with an Si_0.7_Ge_0.3_ buffer layer and its fitting by five Gaussian profiles (*a*); estimated in-plane lattice parameters for similar fittings at different α_i_ values (*b*).

To detect the Si island signal, 2θ/ϕ scans of the 400 reflection were measured at different α_i_ values and analysed by curve fitting. Fig. 7 ▶(*a*) shows one example for the sample with an Si_0.7_Ge_0.3_ buffer layer and Fig. 7(*b*) ▶ a summary of the obtained in-plane lattice parameters. Close to the peak of the Si_0.7_Ge_0.3_ buffer layer, there is another peak, which can be attributed to the Si islands. The misalignment between Si box and Si substrate lattice was relatively low for this sample so that a weak signal of the Si substrate is additionally detected. The averaged in-plane lattice parameter of the Si island signal is *a*_0_ = 5.530 Å, which correlates to a tensile strain of ∊_0_ = 1.8%. The corresponding values for a sample with an Si_0.4_Ge_0.6_ buffer layer are *a*_0_ = 5.513 Å and ∊_0_ = 1.5%, respectively. In combination with specular 004 X-ray diffraction measurements that deliver the off-plane lattice parameter, the real Ge contents for the SiGe buffer layers of the two samples were determined as *x* = 0.51 (instead of 0.30) and *x* = 0.70 (instead of 0.60), respectively. This demonstrates that nanostructured Si islands on SOI substrates require modified recipes compared with bulk Si substrates. Both buffer layers are slightly tensile strained with ∊_0_ = 0.05 and ∊_0_ = 0.13%, respectively. The Ge shows a slightly stronger compressive strain (−0.34%) than in the case of direct deposition without SiGe buffer.

This shows that a significantly stronger compliance effect can be obtained by the use of thin SiGe buffer layers. These results are quite reasonable. The lower misfit between Si and SiGe results in a higher critical thickness for misfit dislocation generation, which allows a stronger strain interaction with the Si islands. For the buffer layer with about 50% Ge, the in-plane lattice parameters of the Si island and SiGe buffer are nearly identical, as demonstrated in Fig. 7 ▶.

**Figure 8:**
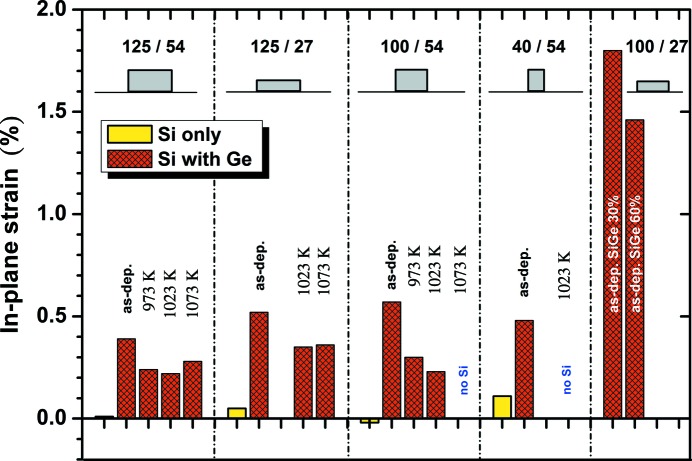
Summary of in-plane strain in Si islands after island preparation only and following Ge deposition for different island geometries and post-annealing.

Fig. 8 ▶ gives a summary of the in-plane strain state of Si nano-islands for different island geometries and preparation processes. It demonstrates again the strong increase of the compliance effect by the use of thin SiGe buffer layers. The use of post-deposition annealing processes, investigated for direct Ge deposition, results in the tendency to reduce the tensile strain in the Si islands. This is probably the result of a further plastic relaxation of the Ge dot structures.

In some cases with small Si island dimensions and/or high annealing temperatures, the Si diffraction signal disappeared completely, which indicates a significant volume reduction by dissolving processes at least below the detection limit.

**Figure 9:**
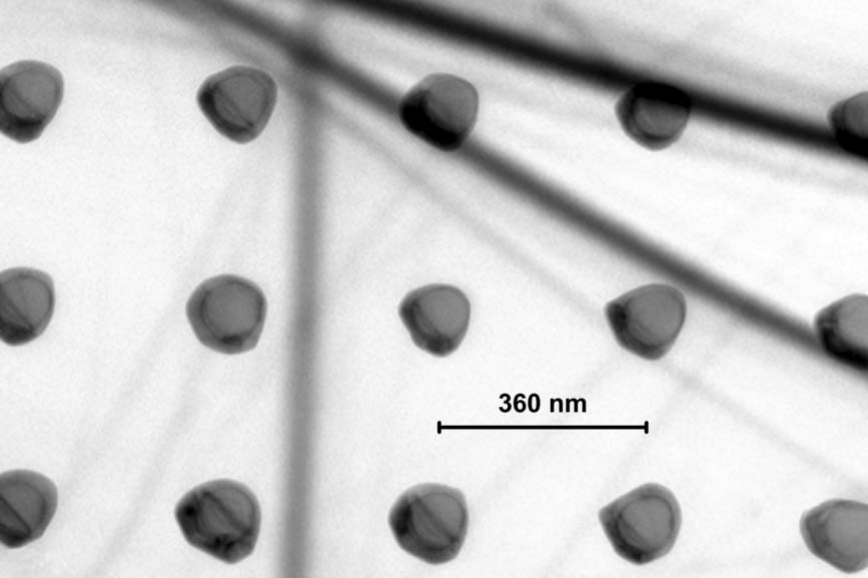
Plane view TEM micrograph of a sample with an Si_0.7_Ge_0.3_ buffer layer showing the Ge/SiGe/Si nano-islands only.

Obviously, the use of a thin SiGe buffer layer leads to a measureable compliance effect in the Si nano-islands. To answer the key question of the structural perfection, and whether the obtained effect is sufficient to avoid the generation of misfit dislocations in the Ge/SiGe/Si material stack, TEM studies were performed. Fig. 9 ▶ shows a TEM micrograph of a sample with an SiGe buffer layer with nominal 30% Ge. Some of the dots with diameter ∼100 nm seem to be free of defects. At least, there is no grid of misfit dislocations visible as was the case for Ge deposited on Si nano-pillars of only 40 nm diameter without an SiGe buffer layer (Kozlowski *et al.*, 2011 ▶). Other dots show structural defects, which are probably stacking faults or micro twins generated mainly at the edge of the Si islands that are overgrown by the Ge (Zaumseil, Kozlowski, Schubert *et al.*, 2012 ▶; Zaumseil, Kozlowski, Yamamoto *et al.*, 2012 ▶).

## 4. Summary and conclusions

We have demonstrated that the strain characterization of nano­structured Si islands on SOI substrates with two-dimensional periodic arrangement is possible by the use of a laboratory-based X-ray diffraction technique. It was con­firmed that the use of about 10 nm-thick Si_(1−*x*)_Ge_*x*_ buffer layers improves the compliance behaviour of the Si islands significantly, up to ∼1.8% tensile strain in one example, and that it seems to be possible to grow Ge on Si free of misfit dislocations in nano-scaled structures. This ‘proof of principle’ study is the basis for further systematic studies to find optimal parameters concerning Si island geometry (diameter, thickness, eventually side wall protection), SiGe buffer parameters (Ge content, thickness) and Ge deposition (growth and annealing temperature, deposition rate *etc*.).

The demonstrated compliant behaviour of Si nano-islands offers a principle way toward Ge/Si nanostructures that are free of misfit dislocations and other structural defects. Therefore it is necessary to use the structural perfection of the fabricated structures as the criterion for the above-mentioned optimization of deposition parameters. X-ray techniques may help in this process, but it requires the complex use of additional techniques like TEM and finally optical and/or electrical methods.

The better understanding of the compliance in Ge/Si nano-heterostructures, as demonstrated in these studies, can furthermore pave the way to integrate not only high-quality Ge but also III–V and II–VI nanostructures on Si.
